# Supplementary material for: Risk Factors and Outcomes of Patients Colonized with KPC and NDM Carbapenemase-Producing Enterobacterales
Source: Antibiotics (Basel). 2024 May 8;13(5):427. doi: 10.3390/antibiotics13050427 (PMC11117268; doi:10.3390/antibiotics13050427)
Supplement: Supplementary file 1 [file antibiotics-13-00427-s001.zip › antibiotics-2940764-supplementary.pdf]

Supplementary Table

Table S1. Charlson Comorbidity Index co-morbidities; KPC-CPE colonized patients vs. NDM-CPE colonized patients.

Variable	KPC-CPE n=92	NDM-CPE n=136	p-value	OR [95% CI]
Myocardial infarction	54	30	<0.001	5 [2.8 – 8.9]
Congective heard failure	12	24	0.459	
Peripheral vascular disease	14	7	0.017	3.3 [1.3 – 8.5]
Cerebrovascular accident or transient ischemic attack	14	13	0.217	
Dementia	20	7	<0.001	5 [2 -12.6]
Chronic obstructive pulmonary disease	9	20	0.315	
Connective tissue disease	2	5	0.704	
Peptic ulcer disease	1	2	1	
Liver disease	8	0	<0.001	undefined
Diabetes mellitus	35	42	0.32	
Hemiplegia	13	10	0.12	
Moderate to severe chronic kidney disease	18	21	0.476	
Solid tumor	7	11	1	
Leukemia	2	0	0.163	
Lymphoma	3	5	1	
AIDS	1	0	0.406	
